# The insufficiency of circulating miRNA and DNA as diagnostic tools or as biomarkers of treatment efficacy for *Onchocerca volvulus*

**DOI:** 10.1038/s41598-020-63249-4

**Published:** 2020-04-21

**Authors:** Cara L. Macfarlane, Shannon Quek, Nicolas Pionnier, Joseph D. Turner, Samuel Wanji, Simon C. Wagstaff, Mark J. Taylor

**Affiliations:** 1 Centre for Drugs and Diagnostics, Department of Tropical Disease Biology, Liverpool School of Tropical Medicine, Liverpool, United Kingdom; 2 Research Foundation for Tropical Diseases and the Environment (REFOTDE) and Department of Microbiology and Parasitology, University of Buea, Buea, Cameroon

**Keywords:** Biomarkers, Diseases

## Abstract

Skin snip evaluation for onchocerciasis has insufficient sensitivity when skin microfilarial (mf) densities are low, such as following ivermectin treatment. Mf density is suitable for assessing microfilaricidal efficacy but only serves as an indirect indicator of macrofilaricidal activity. We assessed circulating nucleic acids from *Onchocerca volvulus* as an alternative to skin snips. We screened a plasma sample set of infected individuals followed up at four, 12 and 21 months after microfilaricidal (ivermectin, n = four), macrofilaricidal (doxycycline, n = nine), or combination treatment (n = five). Two parasite-derived miRNAs, cel-miR-71-5p and bma-lin-4, and O-150 repeat DNA were assessed. Highly abundant DNA repeat families identified in the *O. volvulus* genome were also evaluated. miRNAs were detected in two of 72 plasma samples (2.8%) and two of 47 samples (4.3%) with microfilaridermia using RT-qPCR. O-150 DNA was detected in eight (44.4%) baseline samples by qPCR and the number of positives declined post-treatment. One doxycycline-treated individual remained O-150 positive. However, only 11 (23.4%) samples with microfilaridermia were qPCR-positive. Analysis by qPCR showed novel DNA repeat families were comparatively less abundant than the O-150 repeat. Circulating parasite-derived nucleic acids are therefore insufficient as diagnostic tools or as biomarkers of treatment efficacy for *O. volvulus*.

## Introduction

Onchocerciasis, or “river blindness” is a parasitic disease caused by the filarial worm *Onchocerca volvulus*. Over 100 million people are at risk of infection, of which 99% reside in 31 sub-Saharan African countries endemic for onchocerciasis^1,2^. In Africa, the standard elimination strategy consists of annual mass drug administration (MDA) with ivermectin (Mectizan; IVM). IVM kills microfilariae (mf) in the skin and is used in MDA programmes, which aim to reduce disease burden and block transmission to black fly vectors (*Simulium* spp.)^3^.

A specific and sensitive diagnostic test is needed for onchocerciasis ‘end-game’ scenarios, both to verify elimination and to detect cases when endemicity levels no longer justify MDA^4^. High sensitivity is required for *O. volvulus* hypoendemic areas to detect low mf densities, as well as occult and amicrofilaridermic infections, and to monitor infection recrudescence^5^. High specificity is also required to discriminate between closely related filarial nematodes with overlapping geographic distributions. This is particularly relevant in areas co-endemic for *O. volvulus* and the filarial worm *Loa loa*, such as in “hypoendemic hotspots”^6^, where IVM treatment can cause serious adverse events (SAEs) when *L. loa* microfilaraemia is high (>30,000 mf/ml)^7^. In these areas, alternative strategies with drugs that are safe to use with loiasis will be required to meet elimination targets^8^. Alternative ‘test and (not) treat’ (TaNT) approaches, Loa-first and Oncho-first, can be used to identify and exclude people at risk of SAEs or those not infected with onchocerciasis^9^. While the new rapid LoaScope can identify high *L. loa* microfilaraemia^10,11^, a caveat to strategies based on diagnosing onchocerciasis prior to treatment is the lack of a sufficiently sensitive diagnostic test.

The recent World Health Organization (WHO) guidelines for stopping MDA and verifying onchocerciasis elimination^4^ recommend serological evaluation in sentinel populations by anti-*Ov*16 ELISA, pending validation of the anti-*Ov*16 rapid diagnostic test (RDT) for field use^12^. Both immunoassays detect IgG4 antibodies to the *O. volvulus* antigen *Ov*16, and immunodiagnosis in children can confirm infection exposure and ongoing transmission^4^. However, serological testing cannot distinguish between historical and patent infection. Furthermore, there are concerns over the sensitivity of the *Ov*16 RDT in hypoendemic areas and areas with low or suppressed transmission^13^.

Historically, the ‘gold standard’ diagnostic test used skin snips and morphological evaluation of emergent mf by microscopy. However, this is not recommended by the WHO as it has reduced sensitivity when mf densities are low^14^. To increase sensitivity, skin snips can be analysed for *Onchocerca* genus-specific 150 bp tandem repeat sequence using PCR^15,16^. An *O. volvulus*-specific probe can achieve species-specificity^17^. Real time PCR assays and loop mediated isothermal amplification (LAMP) methods have also been developed for amplification of *O. volvulus* DNA targets^18–22^. However, molecular testing still depends on detection of *O. volvulus* DNA in the skin, and so may be unreliable following treatment or with amicrofilaridermic infections. Skin snips are also invasive and becoming increasingly unpopular, and the procedure is often refused by endemic communities^23^.

A potential alternative is the detection of circulating *O. volvulus* DNA, where sample collection is less invasive, and diagnosis does not rely on the temporal presence of microfilaridermia. A recent study compared parasitological evaluation to high resolution melting (HRM) qPCR for detection of *Brugia malayi* from infected cat blood before and up to two years after receiving either doxycycline (DOXY), IVM or a combination treatment^24^. The qPCR assay positively identified infected cats that were amicrofilaraemic by microscopy, and this test was more sensitive in all groups up to 5–8 months post-treatment^24^. Onchocercomas, the subcutaneous nodules containing adult worms, are highly vascularised^25,26^, and so *O. volvulus* DNA may also be detectable in the blood using a sensitive qPCR platform. PCR has been used to detect O-150 repeats from *Onchocerca gibsoni* in cattle serum^27^; however, to the best of our knowledge, PCR has only been used to test skin snips for human onchocerciasis. Detection of circulating *O. volvulus* DNA could enable diagnosis of infection irrespective of mf status and may provide an indication of infection intensity. The true genome-wide copy number of the O-150 repeats was recently estimated at 5,920 from the new high-quality genome assembly of *O. volvulus*^28^ (previous estimates were around 4000 copies^16^). This genome assembly may also facilitate the identification of novel and potentially more abundant *O. volvulus* DNA repeat families, which could be exploited as diagnostic targets.

Another possibility for *O. volvulus* diagnosis is the potential presence of circulating parasite-derived microRNAs (miRNAs). RNA-based methods may be particularly useful for filarial infections by facilitating identification of species- and stage-specific expression^29,30^. miRNAs are small (~22 nt in length) non-coding RNAs that function as post-transcriptional gene regulators^31^. miRNAs are also present in mammalian extracellular body fluids such as plasma, where they are believed to be particularly stable^32,33^. miRNAs from parasitic nematodes and trematodes are known to be secreted/excreted into their hosts^34–40^, and a preliminary study showed that miRNAs from *Schistosoma mansoni* enabled differentiation between uninfected and infected human serum^36^. Although several studies have confirmed the conserved nature of miRNA secretion/excretion by nematodes^34,37–39,41^, verifying the presence of conserved circulating *O. volvulus* miRNAs would demonstrate the potential of miRNAs for onchocerciasis diagnosis or as a biomarker of treatment efficacy.

The aim of this study was to assess the potential of circulating *O. volvulus* nucleic acids for diagnosis of infection and antifilarial treatment efficacy. A longitudinal plasma sample set collected during a randomised intervention trial enrolling infected individuals in Cameroon^42^ was screened pre- and post-treatment. Patients received either macrofilaricidal (DOXY), microfilaricidal (IVM) or combination treatment. We report here the use of RT-qPCR to detect *O. volvulus* miRNAs lin-4 and miR-71, selected as they have been sequenced in plasma from infected individuals in Cameroon^39^, and qPCR to detect O-150 repeat DNA in plasma. We further investigated additional novel *O. volvulus* DNA repeat families identified through bioinformatic approaches as alternative targets to the O-150 sequence.

## Methods

### Study design and participants

Plasma samples were collected as part of a randomised community-based trial registered with the current controlled trials registry (trial registry no. ISRCTN48118452)^42^. The trial experimental protocol was designed in accordance with the general ethical principles outlined in the Declaration of Helsinki. The trial was approved by ethics committees of the Tropical Medicine Research Station, Kumba and the Research Ethics Committee of The Liverpool School of Tropical Medicine. Written informed consent was obtained from all participants, with the exception of those who were illiterate, where a literate witness signed on behalf of the participant and the participant added a thumbprint.

The double-blind, randomised field trial was undertaken in six satellite villages (Bifang, Ebendi, Eka, Ngalla, Dinku and Olurunti) in the North West Province of Cameroon, starting on 1 July 2003 and finishing on 31 March 2005^42^. All enrolled individuals (adults of both sexes aged 15–60) had *O. volvulus* microfilaridermia >10 mf/snip and were assigned to one of three drug regimens:

1. DOXY: Doxycycline for six weeks plus dummy pill at month four.
2. DOXY + IVM: Doxycycline for six weeks plus ivermectin at month four.
3. IVM: Dummy pill for six weeks and ivermectin at month four.

Plasma and skin snips were collected from participants prior to first treatment and at sequential time points (four, 12 and 21 months) after initiating the study. Two skin snip samples of approximately 1 mg were taken from the rear of the leg using a Walser skin punch. Skin snips were placed in 200 µl saline containing 2 mM EDTA and incubated overnight at room temperature. *O. volvulus* microfilaridermia and *L. loa*/*Mansonella perstans* microfilaraemia were assessed by microscopy at each time point.

For the purposes of this study we omitted participants with filarial co-infections, so the final sample-set included: DOXY (n = 9), DOXY + IVM (n = 5) and IVM (n = 4). The median age was 30, 40 and 42.5 years for the DOXY, DOXY + IVM and IVM groups, respectively. The ratio of female to male participants was 1:1, and evenly distributed between groups. Individual participant characteristics and parasitological evaluation are provided in Supplementary Table S1. European control plasma was obtained from the national health service, UK. All plasma samples were stored at −80 °C.

### miRNA extraction

The Research Foundation for Tropical Diseases and the Environment (REFOTDE), Cameroon provided adult male *Onchocerca ochengi* and L4 *L. loa*. RNA was obtained using the miRCURY RNA Isolation Kit – Cell and Plant (Exiqon, Denmark), following the manufacturer’s instructions. Briefly, two worms were homogenised in lysis buffer using the MagNA Lyser (Roche Diagnostics Ltd, UK) at speed 4000 for 30 sec. DNase treatment was performed. Total RNA was eluted into 50 μl of Elution Buffer and stored at −80 °C. RNA and absence of DNA was quantified using Qubit RNA BR Assay Kit and Qubit dsDNA HS Assay Kit, respectively, for Qubit 3.0 Fluorometer (ThermoFisher, UK).

RNA was extracted from plasma using the miRCURY RNA Isolation Kit – Biofluid (Exiqon, Denmark), following the manufacturer’s instructions with minor modifications. Briefly, 300 µl of plasma was centrifuged at 16 000 × g for 5 min to pellet insoluble material, and 200 µl supernatant removed for extraction. MS2 carrier RNA (Qiagen, UK) and an RNA spike-in mixture (Exiqon, Denmark), consisting of a high, medium and low abundance synthetic miRNA, was added to monitor technical reproducibility and extraction efficiency. DNase treatment was performed. Small RNA (<1000 nt) was eluted into 50 µl RNase free H_2_O and stored at −80 °C. RNA used in downstream applications was based on sample input.

### miRNA primers and primer validation

miRNAs were reverse transcribed using the Universal cDNA Synthesis Kit II (Exiqon, Denmark). Locked Nucleic Acid-enriched miRNA-specific qPCR primers were obtained from Exiqon (Denmark).

Parasite miRNAs lin-4 and miR-71 are here referred to as bma-lin-4 (accession no. MI0013320) and cel-miR-71-5p (accession no. MIMAT000003), respectively, following the miRBase^43^ (release 21) naming convention. Assay efficiencies were determined using five log_10_ serial dilutions of *O. ochengi* cDNA, performing each dilution in triplicate. Experiments were repeated three times to calculate inter- and intra-assay coefficient of variation (CV). To determine the assay limit of detection (LOD), cel-miR-71-5p and bma-lin-4 PCR products were purified using the QIAquick PCR Purification Kit (Qiagen, UK). miRNA amplicon stocks were quantified using Qubit 3.0 Fluorometer (ThermoFisher, UK) and copy numbers were determined with Science Primer^44^. A 1:10 dilution series spanning 10^5^ to 10^0^ copies was prepared for both cel-miR-71-5p and bma-lin-4, with nine reactions per dilution. The 95% LOD was determined by probit regression analysis (SPSS, Version 23, IBM Corp). Assay specificity was evaluated by melt curve analysis, and by using European control plasma and ‘no template control’ (NTC) reactions.

Six additional miRNA qPCR assays were tested to identify a suitable human reference gene: hsa-miR-16-5p, hsa-miR-103a-3p, hsa-miR-425-5p, hsa-miR-93-5p, hsa-miR-191-5p and hsa-miR-484. For hsa-miR-16-5p (accession no. MIMAT0000069), assay efficiency and reproducibility were determined as described above using plasma cDNA. A qPCR assay for UniSp5, the low abundance synthetic spike-in miRNA, was also obtained for experiments.

### miRNA RT-qPCR

The miRCURY LNA Universal RT microRNA PCR (Exiqon, Denmark) was used for miRNA RT-qPCR. For cDNA synthesis, reactions were prepared to 10 µl final volumes following the manufacturer’s instructions. RNA input volume was optimised for clinical samples. Total RNA from *O. ochengi* was added at 10 ng per reverse transcription (RT) reaction. A ‘no reverse transcriptase’ (-RT control), substituting enzyme mix with nuclease-free H_2_O, was prepared for each sample. RT was conducted using the TC-4000 Thermal Cycler (Bibby Scientific Ltd, UK) with thermocycling parameters: 42 °C for 60 min, 95 °C for 5 min, and a cool down to 4 °C.

qPCR was performed in 10 µl reaction volumes, consisting of: 2x ExiLENT SYBR Green Master Mix (Exiqon, Denmark), primer mix (1 µl) and cDNA template (4 µl). cDNA from the worms was diluted to the desired concentration and dilution volumes were optimised for clinical samples. The CFX384 C1000 Thermal Cycler (Bio-Rad, UK) was used for qPCR, with thermocycling parameters: 95 °C hold for 10 min, 40 cycles of 95 °C for 10 sec, and 60 °C for 1 min with a ramp rate of 1.6 °C/sec. Fluorescence was monitored during the 60 °C step, using the FAM channel. Melt curve analysis was performed between 60 and 95 °C at a ramp rate of 0.5 °C/sec. For each experiment, samples were tested in duplicate reactions and a positive control, NTC and -RT control (for each sample) was included.

Samples were considered positive if amplification occurred in fewer than 40 cycles and in both reactions. A single peak at the correct melting temperature (T_m)_ was required for each product. The T_m_ was determined from standard curves prepared from *O. ochengi* cDNA. Samples positive in one reaction were retested in triplicate and considered positive if amplification occurred in two or more reactions.

### DNA extraction

*O. volvulus* DNA had previously been extracted from a human onchocercoma and skin snip collected between 2003–2005 during the trial in Cameroon^42^. DNA was stored at −80 °C.

Plasma DNA was extracted using the QIAamp DNA Blood Mini Kit (Qiagen, UK) following the manufacturer’s instructions with minor modifications. Briefly, 300 µl of plasma was centrifuged at 16 000 x g for 5 min and 200 µl supernatant was collected for DNA extraction. To control for technical variation, phocine herpes virus-1 (PhHV-1) DNA (Clinical Virology Department, Erasmus MC, Netherlands) was diluted 1:1000 in nuclease-free H_2_O and spiked at 1 µl/200 µl lysis buffer. DNA was eluted into 50 µl Buffer AE and stored at −80 °C. DNA input in downstream applications was based on initial sample volume.

### Novel DNA targets: repeat region identification

A species-specific repeat library of *O. volvulus* was generated using RepeatModeler (Version 1.0.11)^45^ with default parameter settings. This repeat library was used, alongside a general repeat library from RepBase (RepBase RepeatMasker edition 20170127)^46^, as a filter against the *O. volvulus* genome^28^ using RepeatMasker (Version 4.0.8)^47^. This generated a list of repeat families and their predicted location, number of occurrences and total genome coverage.

The five most abundant repeat families and five abundant repeat families that shared one or more contigs with the O-150 repeat region were selected for further investigation. Specificity was confirmed via BLAST searches of the *Onchocerca genus, L. sigmondontis* and *L. loa* genome repositories and the *M. perstans* nucleotide repository.

### Primers and probes for DNA-based experiments

Assay sequences are provided in Supplementary Table S2.

A TaqMan qPCR assay^19^ (IDT, Belgium) was used to detect the O-150 sequence (accession no. J04659). Details of assay design are reported elsewhere^19^. Assay linearity was assessed from standard curves prepared from five 1:2 serial dilutions of *O. volvulus* DNA, with three replicates per dilution. Experiments were repeated three times to determine intra- and inter-assay CV. The assay LOD is reported elsewhere^19^. Assay specificity was confirmed using control plasma and NTCs.

A qPCR assay (Sigma) for the plasma endogenous control glyceraldehyde 3-phosphate dehydrogenase (GAPDH) (accession no. NM_002046.5) was assessed as described above using plasma DNA. A TaqMan assay for viral DNA spike-in PhHV-1 (accession no. Z68147.1) was purchased from IDT (Belgium).

Up to three primer sets were designed for each novel repeat family using Primer-BLAST^48^. Primers were validated by testing *O. volvulus* DNA in duplicate reactions. Standard curves were prepared for primers with a quantification cycle (Cq) value within 6.6 Cqs of the O-150 repeat’s Cq value, using six 1:10 serial dilutions of *O. volvulus* DNA and three replicates per dilution.

### qPCR for DNA-based experiments

All experiments were performed with the CFX384 C1000 Thermal Cycler (Bio-Rad, UK).

#### Evaluation of O-150 DNA

Reaction mixes were prepared to 20 μl final volumes. O-150 qPCR reactions included: 2X TaqMan Fast Advanced Master Mix (Thermo Scientific), 300 nM primers, 250 nM probe, nuclease-free H_2_O and 4 μl DNA. PhHV-1 reactions contained: 2X TaqMan Fast Advanced Master Mix (Life Technologies), 100 nM primers and probe, nuclease-free H_2_O and 4 μl DNA. qPCR cycling parameters are reported elsewhere^19^. Fluorescence was monitored using the FAM channel and VIC channel for O-150 and PhHV-1, respectively.

GAPDH qPCR reaction mixtures consisted of: 2X SsoAdvanced Universal SYBR Green Supermix (Bio-Rad, UK), 400 nM primers, nuclease-free H_2_O and 4 μl DNA. Cycling parameters were: a hold at 98.0 °C for 3 min, and 40 cycles of 98.0 °C for 10 sec and 60 °C for 20 sec. Fluorescence was monitored during the 60 °C step using the FAM channel. Melt curve analysis was performed between 60 and 95 °C at a ramp rate of 0.5 °C/s.

Experiments included a positive control and NTC. Samples were verified as positive using the same criteria described for miRNA samples, minus the melt curve analysis.

#### Evaluation of novel repeat family DNA

Primer evaluation was performed in 20 μl final volumes. Experiments involving plasma DNA were performed in 10 μl final volumes due to sample limitations. Reaction mixes consisted of: Quantitect SYBR Green Mix (Qiagen, UK), 300 nM primers, nuclease-free H_2_O and 2 μl of DNA.

Cycling parameters consisted of: a hold at 95 °C for 15 min, 40 cycles of 94 °C for 15 sec and 57 °C for 30 sec, and extension at 72 °C for 30 sec. Fluorescence was monitored during the 72 °C step, using the FAM channel. Melt curve analysis was performed between 60 °C to 97 °C at a ramp rate of 0.2 °C/sec.

### Statistical analysis

Probit regression analysis was performed in SPSS (Version 23, IBM Corp). As sample sizes were unequal and small, non-parametric statistical analyses were performed in GraphPad Prism 5^49^. Levels of plasma reference targets were compared between treatment groups at each time point using Kruskal-Wallis one-way ANOVA. The consistency of endogenous control levels within treatment groups over time was assessed by Friedman’s test, followed by Dunn’s post hoc test (with 95% confidence intervals). Significance level was set to alpha = 0.05.

## Results

### Parasite miRNA RT-qPCR

The parasite miRNAs bma-lin-4 and cel-miR-71-5p were detected in RNA extracted from both adult male *O. ochengi* and L4 *L. loa* worms. The miRNA assays tested negative in European control plasma and in NTC and -RT control reactions. The efficiency of the cel-miR-71-5p qPCR assay was 99.9%, with an R^2^ value of 0.979. Melt analysis detected a single peak at a T_m_ of 69.5 °C. The bma-lin-4 qPCR assay efficiency was 97.3%, and the R^2^ was 0.993. Melt analysis detected a single peak at a T_m_ of 71.5 °C. Further details of assay validation, including the inter- and intra-assay CV, the average qPCR efficiency across experiments, the linear dynamic range and the LOD, are provided with Supplementary Fig. S1.

Six human extracellular miRNAs (hsa-miR-16-5p, hsa-miR-103a-3p, hsa-miR-425-5p, hsa-miR-93-5p, hsa-miR-191-5p and hsa-miR-484) were evaluated to identify a reference control. Standard curves were prepared for hsa-miR-16-5p as this was the most abundant miRNA in plasma. The efficiency of the hsa-miR-16-5p qPCR assay was 106.7%, with an R^2^ of 0.990. Assay evaluation and validation details are provided in Supplementary Fig. S2.

### Detection of parasite miRNAs in clinical plasma samples

RNA and cDNA inputs for RT and qPCR, respectively, were determined empirically by evaluating several RNA input volumes and cDNA dilutions (Supplementary Fig. S3). Plasma from 18 microfilaridermic individuals was screened for the presence of *O. volvulus* miRNAs at baseline and at four, 12 and 21 months post-treatment. Two of the 72 plasma samples (2.8%) and two of the 47 samples with microfilaridermia (4.3%) were very weakly positive for a parasite miRNA. Cel-miR-71-5p was detected in one DOXY patient (patient *a.5*) with 2 mf/snip at month four; the average Cq and standard deviation (± SD) from three replicates was 38.02 ± 0.339. Bma-lin-4 was detected in one DOXY patient (patient *a.7*) with 1 mf/snip at month 12; the average Cq from three replicates (± SD) was 36.75 ± 0.212. All control reactions were negative.

The endogenous control miRNA hsa-miR-16-5p was consistently detected (Supplementary Fig. S4) and there was no significant difference in expression levels between groups at any time point. The IVM and DOXY + IVM groups also showed no significant change in expression in individuals over time. However, a significant difference was detected within the DOXY group over time (Friedman’s test: P = 0.0191). Dunn’s post hoc test indicated the difference was between months four and 12 (P < 0.05). Hsa-miR-16-5p, and to a lesser extent the spike-in UniSp5, had a higher average Cq value in samples at month four relative to month 12. This is likely due to a small degree of inhibition. Overall, the synthetic low abundance miRNA spike-in UniSp5 was consistently and comparably detected across all samples (Supplementary Fig. S4).

### *Onchocerca volvulus* DNA qPCR

We next sought to establish whether *O. volvulus* DNA could be detected in host plasma. The O-150 TaqMan assay was evaluated by creating standard curves; the efficiency was 97.7% with an R^2^ value of 0.964. The O-150 assay was negative in control plasma and NTC reactions. The efficiency of a qPCR assay for the plasma endogenous control GAPDH was 100.1%, with an R^2^ value of 0.944. Further details of O-150 and GAPDH assay validation are provided with Supplementary Fig. S5.

### Detection of O-150 DNA in clinical plasma samples

Of the 18 trial participants evaluated with the O-150 assay, eight (44.4%) were positive for O-150 at baseline, four (22.2%) were positive at month four, and one (5.6%) was positive at months 12 and 21 (Table 1). In each treatment group, the proportion of O-150 positive patients was higher at baseline and at month four. Four months into the trial, the number of positive patients had declined from four to one (75% decrease) with DOXY, and three to one (66.7% decrease) with DOXY + IVM. The number of O-150 positive individuals initially increased in the IVM group from one to two (50% increase), but IVM was not provided until month four of the trial. Two individuals, one treated with DOXY + IVM and one with IVM, were positive at both baseline and month four, but negative at later time points. One individual treated with DOXY, who was also positive for miRNA bma-lin-4, remained O-150 positive over the trial duration.

**Table 1:** Comparison of plasma qPCR and skin snip parasitological evaluation.

		Baseline		Month four		Month 12		Month 21	
Treatment	Patient	qPCR	mf/snip	qPCR	mf/snip	qPCR	mf/snip	qPCR	mf/snip
**DOXY**	*a.1*	Neg	40	Neg	1	Neg	2	Neg	Neg
	*a.2*	39.26 ± 1.24	15.5	Neg	34	Neg	6.5	Neg	Neg
	*a.3*	37.28 ± 1.05	14	Neg	2	Neg	Neg	Neg	Neg
	*a.4*	38.22 ± 1.24	20.5	Neg	10	Neg	Neg	Neg	Neg
	*a.5*	Neg	16.5	Neg	2	Neg	Neg	Neg	3.5
	*a.6*	Neg	23.5	Neg	1	Neg	Neg	Neg	8.5
	*a.7*	34.31 ± 0.54	13	36.88 ± 0.44	Neg	38.43 ± 1.02	1	37.47 ± 0.65	Neg
	*a.8*	Neg	19	Neg	93	Neg	11.5	Neg	Neg
	*a.9*	Neg	45	Neg	6	Neg	Neg	Neg	Neg
**DOXY** + **IVM**	*b.1*	Neg	18	Neg	9	Neg	1.5	Neg	Neg
	*b.2*	Neg	24	Neg	1	Neg	Neg	Neg	Neg
	*b.3*	38.79 ± 0.52	34.5	Neg	13	Neg	0.5	Neg	Neg
	*b.4*	38.37 ± 1.66	88	38.61 ± 0.84	Neg	Neg	Neg	Neg	1
	*b.5*	38.61 ± 0.46	61.5	Neg	28	Neg	Neg	Neg	Neg
**IVM**	*c.1*	38.26 ± 0.26	90.5	36.03 ± 0.38	99	Neg	0.5	Neg	Neg
	*c.2*	Neg	12	Neg	Neg	Neg	Neg	Neg	0.5
	*c.3*	Neg	15.5	Neg	1	Neg	Neg	Neg	2
	*c.4*	Neg	23.5	38.42 ± 0.99	2	Neg	30	Neg	8.5

Trial participants screened longitudinally for circulating O-150 DNA by qPCR and their corresponding average mf/snip (mf/mg) are detailed. Mean Cq value and ± SD for positive results are displayed. Cq, quantification cycle; mf, microfilariae; Neg, negative; SD, standard deviation.

Of the 72 samples evaluated, 47 were microfilaridermic and 14 were O-150 positive. Among the samples with microfilaridermia, only 11 (23.4%) were O-150 positive. Although plasma qPCR did identify three infected but amicrofilaridermic individuals, plasma qPCR did not detect a considerable proportion of the infected individuals, many of whom had low infection intensities. Around 1/3 of samples were negative by both qPCR and skin snip.

At baseline, most individuals positive by qPCR had similar mf densities to the qPCR negative patients, with the average mf/snip ranging from 13 - 90.5 in O-150 positive patients and from 12–45 among the negative patients. Similarly, levels of O-150 DNA did not appear to correlate with mf density, as patient *a.7* had the lowest mf density and highest relative levels of O-150. At month four, there was also little correlation between the frequency of O-150 positives or O-150 DNA levels and mf intensity.

The endogenous control GAPDH was detected in all samples, and relative levels did not differ significantly between or within treatment groups over time. The viral control spike-in PhHV-1 confirmed DNA was extracted uniformly across all samples. This data is shown in Supplementary Fig. S6.

### Identification and validation of novel DNA repeat families

RepeatModeler^45^, RepBase datasets^46^ and RepeatMasker^47^ were used to bioinformatically predict repeat regions and families in the *O. volvulus* genome. This identified 259 repeat families that are not in publicly available databases, collectively occupying 7.19% of the *O. volvulus* genome (Supplementary Table S3). While the O-150 repeat region was predicted to occur 46 times, several repeat families were predicted to occur over 1,000 times.

We evaluated five highly abundant families (families A, B, C, D, E) and an additional five families (families F, G, H, I, J) located on contigs shared with the O-150 repeat (Supplementary Table S4). Up to three primer sets were designed for each of the ten repeat families, with primers targeting different regions where possible. The novel repeats were evaluated alongside the O-150 repeat to compare the relative abundance in *O. volvulus* DNA (Table 2). The average Cq value (± SD) was 20.60 (± 0.03) for O-150, and the 10 repeat families ranged from 24.95 (± 0.08) to 34.00 (± 0.14). We planned to optimise five primer sets, but primer efficiencies ranged between 97.1–110.9%, and so optimisation was unlikely to considerably improve assay performance. Contrary to our bioinformatic predications, the novel targets were comparatively less abundant than O-150 in the *O. volvulus* genome.

**Table 2:** Novel DNA repeat families of *Onchocerca volvulus*.

Repeat family	Predicted occurrences	Primer set	Cq value ± SD	% Efficiency	R^2^
**O-150**	46	NA	20.60 ± 0.03	95.1	0.991
**A**	970	I	26.18 ± 1.04	**104.0**	**0.975**
		II	25.37 ± 0.74	**97.1**	**0.994**
		III	24.95 ± 0.08	**100.6**	**0.997**
**B**	1,000	I	27.09 ± 0.04	**110.9**	**0.992**
		II	28.27 ± 0.01	NA	NA
		III	27.41 ± 0.02	NA	NA
**C**	565	I	34.00 ± 0.14	NA	NA
		II	29.09 ± 0.08	NA	NA
		III	28.815 ± 0.12	NA	NA
**D**	1,402	I	29.99 ± 0.10	NA	NA
		II	29.18 ± 0.00	NA	NA
		III	29.745 ± 0.71	NA	NA
**E**	823	I	32.69 ± 0.05	NA	NA
		II	27.27 ± 0.09	NA	NA
		III	29.08 ± 0.01	NA	NA
**F**	172	I	NA	NA	NA
		II	32.15 ± 0.16	NA	NA
		III	27.18 ± 0.05	NA	NA
**G**	252	I	NA	NA	NA
		II	26.01 ± 0.11	**106.1**	**0.996**
**H**	100	I	NA	NA	NA
		II	NA	NA	NA
**I**	102	I	32.95 ± 0.09	NA	NA
		II	28.36 ± 0.24	NA	NA
**J**	196	I	28.29 ± 0.05	NA	NA

The Cq values and ± SD of all primer sets for *O. volvulus* repeat families. The efficiency and R^2^ of standard curves are shown for five assays that had a Cq value within 6.6 Cqs of the O-150 assay Cq value. Cq, quantification cycle; NA, not applicable; SD, standard deviation.

### Detection of novel DNA repeat families in clinical plasma samples

To determine whether the new *O. volvulus* targets were detectable in plasma, primers for repeat families A (primer set A-III) and G (primer set G-II) were evaluated with a random selection of nine of the 18 baseline plasma samples (participants: *a.2, a.6, a.7, a.9, b.1, b.2, b.4, c.3, c.4*). These two primer sets were selected as they were the best performing amongst the 25 primer sets.

Only one individual (*a.7*) was considered positive (Cq ± SD) for circulating O-150 (32.97 ± 0.73) and repeat family G (36.04 ± 0.28). Due to sample limitations, samples with one amplified reaction could not be retested in triplicate (data provided in Supplementary Table S5). Two patients (*a.2* and *b.4*) positive for O-150 in earlier experiments were not positive using this criterion. Regarding samples with amplification in one reaction, all DNA targets were detected at high Cq values (>34.67), and only two patients (*c.3* and *c.4*) may have been positive for Family A-III or Family G-II, but not O-150.

## Discussion

This study reports the first use of RT-qPCR and qPCR to detect *O. volvulus* miRNAs and DNA, respectively, in plasma from individuals in an onchocerciasis-endemic community before and after macrofilaricidal or microfilaricidal treatment. We confirmed the parasite miRNAs cel-miR-71-5p and bma-lin-4 are expressed in *O. ochengi* and *L. loa*, and therefore they are likely also expressed by *O. volvulus*. However, all but two of the 72 clinical plasma samples tested were negative for parasite miRNAs. By comparison, the O-150 DNA target was detected in almost half of the samples collected at baseline of the trial, and the number of positive patients declined over subsequent sampling timepoints in every treatment group. While this initially indicated O-150 could be a marker of microfilaridermia, a high proportion of samples that were mf-positive by parasitological evaluation were negative by qPCR of plasma. Bioinformatic approaches and qPCR were used to identify and validate novel *O. volvulus* DNA repeat families predicted be more abundant than O-150. However, despite the encouraging bioinformatic predications, all the novel targets were confirmed to be less abundant than O-150 in the *O. volvulus* genome.

While miR-71 and lin-4 have previously been detected in host fluids and linked to several filarial worms, including *O. volvulus, O. ochengi, L. loa, L. sigmodontis, Dirofilaria immitis* and *Brugia pahangi*, as well as helminth extracellular exosomes and vesicles^37,40,41,50^, they were not reliable markers for *O. volvulus* infection. The very low proportion of miRNA-positive samples is likely related to the locality of *O. volvulus* adult and larval stages in the host. For example, studies have identified parasite-derived miRNAs in serum of mice infected by *L. sigmodontis* (adults localised in the pleural cavity with mf in blood)^37^, but they were not identified in the serum of mice infected with *Heligmosomoides polygyrus* (localised in the gut lumen)^37^.

A recent study investigated circulating cel-miR-71-5p and bma-lin-4, as well as 15 other putative *O. volvulus* miRNAs, using RT-qPCR^40^. This study also highlighted that levels of extracellular parasite-derived miRNAs are very low, if at all detectable. As miRNAs from blood-localised parasites appear to be more abundant in host circulation relative to parasites in other tissues^34,37^, circulating miRNAs could have originated from another parasite. We attempted to reduce the risk of *L. loa /M. perstans* co*-*infections by eliminating samples from patients infected with other mf species, although we acknowledge there could be occult infections. The study by Lagatie *et al*.^40^ also highlighted inadequate specificity as a concern due to the detection of worm-derived miRNAs in uninfected samples, as well discrepancy in miRNA qPCR melt curves. By comparison, our study validated worm miRNA assays with relevant biological reference species, and the melt curve for the assays was consistent when tested with *O. ochengi*, *L. loa* and patient plasma cDNA.

Use of an *O. volvulus*-specific DNA assay improved the diagnosis of onchocerciasis cases, identifying 44.4% of individuals who were skin snip positive at baseline. The number of individuals O-150 positive then declined in all groups after first treatment. While more samples from infected participants were correctly identified by testing O-150 DNA relative to parasite miRNAs, the majority of microfilaridermic individuals had plasma samples that were negative by qPCR. Furthermore, the Cq values recorded for most of the O-150 positive samples were very high (>37). We re-tested samples and used robust controls in experiments, including a positive control, a NTC, European control plasma and plasma endogenous control, to ensure that we did not accept false positives and plasma nucleic acids were not degraded or affected by plasma-derived inhibitors. Therefore, although TaqMan and qPCR enable detection of targets with high sensitivity and specificity, the very low levels of circulating *O. volvulus* DNA were not sufficient to detect infection.

We attempted to identify novel and more abundant DNA targets to exploit as circulating diagnostic markers or biomarkers of treatment efficacy. The bioinformatic approach used showed similar results, in terms of overall coverage, to those identified in the original genome sequencing paper^28^, in particular the predicted small frequency of O-150 repeats in the genome. Therefore, a novel repeat region could be more suitable as a diagnostic marker. We were unable to identify a superior repeat family after qPCR analysis of *O. volvulus* DNA and clinical plasma DNA, but we can confirm that the true copy number of O-150 repeats is significantly higher than what is bioinformatically predicted. These predicted values did not include the possibility of collapsed contig repeats, an issue previously noted^28^, and this could affect the predicted repeat frequency of O-150 and novel repeat families. Due to these issues, there is a possibility that a different repeat family with an extremely high ‘true’ occurrence rate (like O-150) may exist, but the information available to this study was insufficient to identify it.

We have demonstrated that *O. volvulus* nucleic acids are variably detectable at low to undetectable concentrations in host plasma. Circulating nucleic acids from *O. volvulus* are therefore insufficient as diagnostic markers for onchocerciasis infection or as biomarkers of treatment efficacy. Among the nucleic acids evaluated the O-150 DNA sequence showed the greatest frequency, although well below the sensitivity of parasitological diagnosis. While we did not identify a more abundant novel DNA target from *O. volvulus*, we acknowledge the possibility that a different highly abundant DNA repeat family may still exist.

## Supplementary information

Supplementary Information.

Supplementary Information2.

## Data Availability

The datasets supporting the conclusions of this article are included within the article and the Supplementary Information.
