# Supplementary material for: The insufficiency of circulating miRNA and DNA as diagnostic tools or as biomarkers of treatment efficacy for Onchocerca volvulus
Source: Sci Rep. 2020 Apr 21;10:6672. doi: 10.1038/s41598-020-63249-4 (PMC7174290; doi:10.1038/s41598-020-63249-4)
Supplement: Supplementary file 1 — Supplementary Information. [file 41598_2020_63249_MOESM1_ESM.docx]

**The insufficiency of circulating miRNA and DNA as diagnostic tools or as biomarkers of treatment efficacy for *Onchocerca volvulus***

Macfarlane CL, Quek S, Pionnier N, Turner JD, Wanji S, Wagstaff SC, Taylor MJ

**Supplementary Table S1.** **Study characteristics.**

Baseline characteristics and parasitological evaluation of the 18 microfilaridermic individuals eligible for longitudinal analysis. The patients allocated treatment group, age and gender, number of nodules at baseline, and mean number of mf/mg skin snip at baseline and at four, 12 and 21 months after trial initiation are detailed. T4, month four; T12, month 12; T21, month 21.

| **Treatment** | **Patient** | **Gender** | **Age** | **Nodules at baseline** | **Baseline mf (mf/mg)** | **T4 mf (mf/mg)** | **T12 mf (mf/mg)** | **T21 mf (mf/mg)** |
| --- | --- | --- | --- | --- | --- | --- | --- | --- |
| **DOXY** | *a.1* | F | 25 | 4 | 40 | 1 | 2 | 0 |
|  | *a.2* | M | 54 | 1 | 15.5 | 34 | 6.5 | 0 |
|  | *a.3* | M | 33 | 0 | 14 | 2 | 0 | 0 |
|  | *a.4* | F | 40 | 0 | 20.5 | 10 | 0 | 0 |
|  | *a.5* | M | 15 | 2 | 16.5 | 2 | 0 | 3.5 |
|  | *a.6* | F | 30 | 0 | 23.5 | 1 | 0 | 8.5 |
|  | *a.7* | M | 41 | 0 | 13 | 0 | 1 | 0 |
|  | *a.8* | F | 20 | 1 | 19 | 93 | 11.5 | 0 |
|  | *a.9* | F | 23 | 0 | 45 | 6 | 0 | 0 |
| **DOXY+IVM** | *b.1* | M | 48 | 0 | 18 | 9 | 1.5 | 0 |
|  | *b.2* | F | 40 | 1 | 24 | 1 | 0 | 0 |
|  | *b.3* | M | 25 | 0 | 34.5 | 13 | 0.5 | 0 |
|  | *b.4* | M | 55 | 1 | 88 | 0 | 0 | 1 |
|  | *b.5* | F | 20 | 0 | 61.5 | 28 | 0 | 0 |
| **IVM** | *c.1* | F | 45 | 4 | 90.5 | 99 | 0.5 | 0 |
|  | *c.2* | M | 25 | 0 | 12 | 0 | 0 | 0.5 |
|  | *c.3* | M | 40 | 1 | 15.5 | 1 | 0 | 2 |
|  | *c.4* | F | 50 | 2 | 23.5 | 2 | 30 | 8.5 |

**Supplementary Table S2. Primer and probe sequences for all DNA-based experiments.**

For experiments comparing the relative levels of the O-150 repeat DNA sequence to novel *O. volvulus* repeat families A - J, only the O-150 primers (without the probe) were used.

GAPDH, Glyceraldehyde 3-phosphate dehydrogenase; PhHV-1, phocine herpes virus-1.

| **Target** | **Primer set** | **Primer and probe sequences** |
| --- | --- | --- |
| **GAPDH** | N/A | F 5′- CCACTCCTCCACCTTTGAC -3′  R 5′- ACCCTGTTGCTGTAGCCA -3′ |
| **PhHV-1** | N/A | F 5′- GGGCGAATCACAGATTGAATC -3′  R 5′- GCGGTTCCAAACGTACCAA 3′  Probe 5′- VIC-TTTTTATGTGTCCGCCACCATCTGGATC-BHQ1 -3′ |
| **O-150** | N/A | F 5′- TCGCCGTGTAAATGTGGAA -3′  R 5′- AACTGATGACCTATGACCCTAATC -3′  Probe 5′- FAM-GGACCCAATTCGAATGTATGTACCCGT-Zen/Iowa Black FQ -3′ |
| **A** | I | F 5′- GCGTTGCTGTAGCTCAGTCT -3′  R 5′- GCTGACCTAGCCGAATCACA -3′ |
|  | II | F 5′- TGTTGCGTCGCTTTGAACTG -3′  R 5′- AGTGGCGCGACATAGTTTCA -3′ |
|  | III | F 5′- GCTACTGTTGCGTCGCTTTG -3′  R 5′- CGGAGTTCCAAGGGTTCCAG -3′ |
| **B** | I | F 5′- TCGCAGAAATGTCGAGGTCA -3′  R 5′- AGGCAATCCAAGACACGACC -3′ |
|  | II | F 5′- CGCCCGTGACAGCTAAAAGT -3′  R 5′- GCAGAAAGTAGGCGTGGTGA -3′ |
|  | III | F 5′- GTCGCAGAAATGTCGAGGTC -3′  R 5′- AAGAGGCAATCCAAGACACGA -3′ |
| **C** | I | F 5′- ACCTCCAATAACTCCGCAAGT -3′  R 5′- CTCCGCCTCCTCACTTACTTT -3′ |
|  | II | F 5′- TTCGAATAGGCGAGTGCTCTT -3′  R 5′- AACTCCGCCTCCTCACTTACT -3′ |
|  | III | F 5′- TCGAATAGGCGAGTGCTCTT -3′  R 5′- AAACTCCGCCTCCTCACTTAC -3′ |
| **D** | I | F 5′- GCTGTGTCGGTGGAAGAACT -3′  R 5′- TAAGAAGGAGGAGCCCCGAA -3′ |
|  | II | F 5′- ACTTTTGCGTTGGTTTCCCG -3′  R 5′- GGAAGACTCCTCAACAGACCG -3′ |
|  | III | F 5′- AAATCCTTTCGGGGCTCCTC -3′  R 5′- ACAAAGCGAGAGTCGACCAA -3′ |
| **E** | I | F 5′- TCAATTAGAGAGATGCGCGGAG -3′  R 5′- AGGTGAGGAGGTGGAGTTTATT -3′ |
|  | II | F 5′- TTAGAGAGATGCGCGGAGGA -3′  R 5′- AGGTGGAGTTTATTGGCTCCG -3′ |
|  | III | F 5′- AATTAGAGAGATGCGCGGAGG -3′  R 5′- AGGAGGTGGAGTTTATTGGCTC -3′ |
| **F** | I | F 5′- GGAGCCTGTCGCTGATGTAA -3′  R 5′- GGCAGCGATCTCTCAACACT -3′ |
|  | II | F 5′- GGGGCGGAGGTTCATACATT -3′  R 5′- AAGAGTTGCAAATGGCCGC -3′ |
|  | III | F 5′- GCGGCCATTTGCAACTCTTTA -3′  R 5′- ATCTCATTGCGCACAAAGCTC -3′ |
| **G** | I | F 5′- GTATTCCGGAGATCGCTGCC -3′  R 5′- CCTCCGCCCCTATCAAGGAT -3′ |
|  | II | F 5′- TGTGCTTCGTTGAGTTTGCTT -3′  R 5′- ATCAGCGACAGGCTCTCCTA -3′ |
| **H** | I | F 5′- TCGGTAAACCGAAATGCTGACA -3′  R 5′- GAGCTTTGTGCGCAATGAGATTA -3′ |
|  | II | F 5′- GTCGGTAAACCGAAATGCTGACA -3′  R 5′- GCTTTGTGCGCAATGAGATTACAA -3′ |
| **I** | I | F 5′- ATTGCTTGATATTGGCCGGGA -3′  R 5′- ATCAGCGACAGGCTCCTACA -3′ |
|  | II | F 5′- TGTGCTTCGTTGAGATTGCTTG -3′  R 5′- ATCAGTGGCAAACGTTCCCG -3′ |
| **J** | I | F 5′- TCCCAGCCAATATCAAGCGA -3′  R 5′- CCAACAGTGGTCCTTTGACGA -3′ |

**Supplementary Fig. S1.** **Parasite miRNA qPCR assay validation.**

**A.** Efficiency and precision of the cel-miR-71-5p qPCR assay tested over five 1:10 dilutions of *O. ochengi* cDNA. The overall inter- and intra-assay coefficient of variation (CV) from three qPCR experiments was 1.87% and 0.15%, respectively. The average (± SD) efficiency of the qPCR across the three experiments was 102.7% (3.41). The linear dynamic range of the assay was over 10^5^ to 10^2^ copies, and the mean quantitation cycle (Cq) value across nine reactions at the last linear point of the curve was 34.13 ± 1.50 (± SD). Probit regression analysis determined the 95% limit of detection (LOD) as 140 copies (95% confidence interval, CI: 95 - 325 copies) of cel-miR-71-5p.

**B.** Efficiency and precision of the bma-lin-4 qPCR assay tested over five 1:10 dilutions of *O. ochengi* cDNA. The overall inter and intra assay CV was 1.76% and 0.09%, respectively. The average (± SD) efficiency of the qPCR across the three experiments was 95.0% (3.08). The linear dynamic range of the bma-lin-4 assay was 10^5^ to 10^2^ copies, with a mean Cq value of 35.30 ± 0.76 (± SD) at the final linear point of the curve. The 95% LOD was 73 copies (95% CI: upper and lower bound could not be determined).


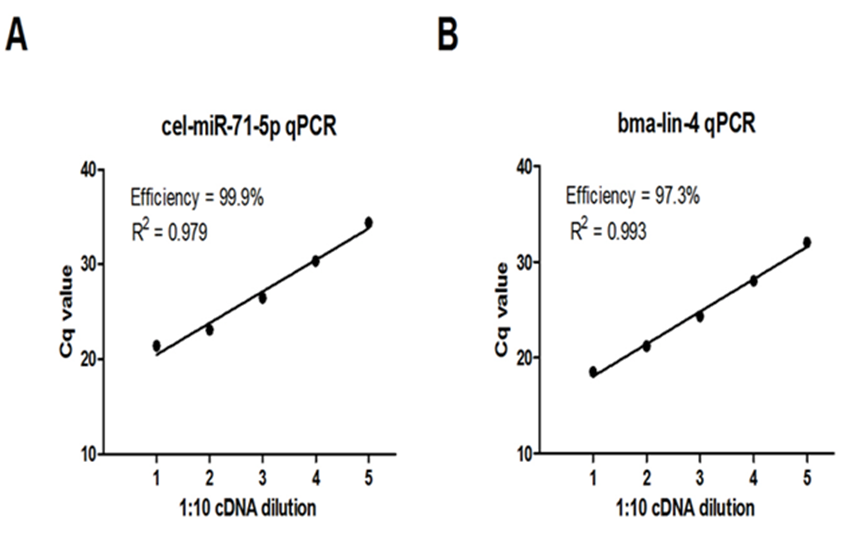


**Supplementary Fig. S2. Assessment of plasma reference miRNA assays.**

**A.** Six miRNA qPCR assays were evaluated to determine a suitable endogenous control for plasma samples. Hsa-miR-16-5p was the most abundant miRNA in European control plasma. For each assay, the mean of three replicates with minimum and maximum values are displayed.

**B.** The hsa-miR-16-5p qPCR assay was tested over five 1:10 dilutions of plasma cDNA. The average of three reactions and ± SD per dilution for each assay is shown. The overall inter- and intra-assay coefficient of variation was 1.34% and 0.04%, respectively. The average (± SD) efficiency of the qPCR across the three experiments was 109.9% (3.20).


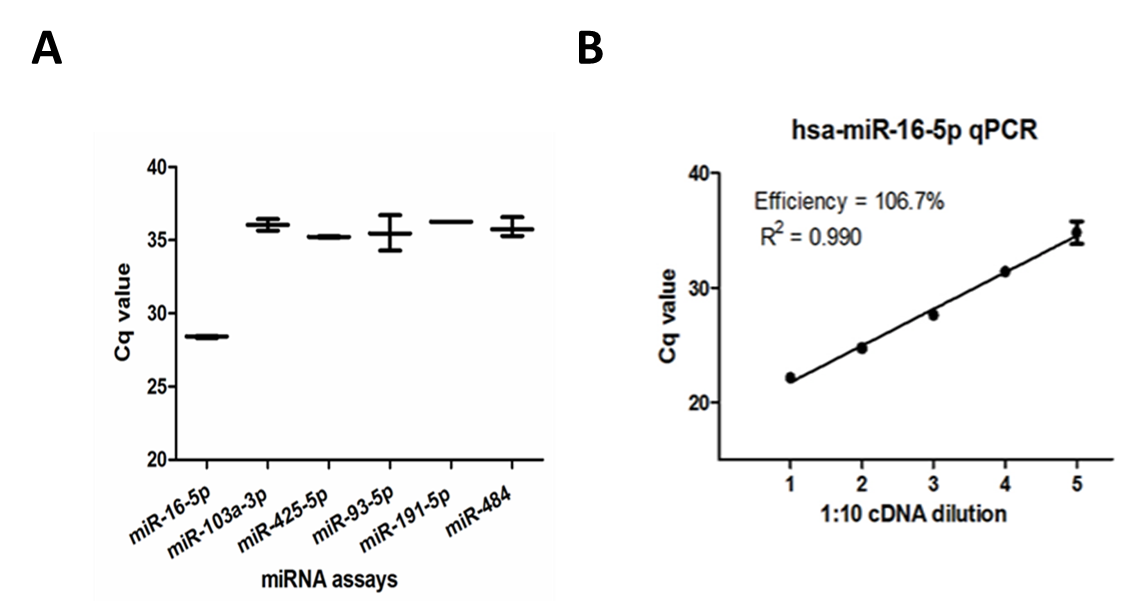


**Supplementary Fig. S3. RT-qPCR optimisation.**

**A-E.** The relative change in Cq value of plasma reference miRNA hsa-miR-16-5p in European control plasma **(A)** and plasma taken at baseline from four individuals from the trial (**B-E**). Inhibition was not detected in the trial samples after increasing RNA and cDNA inputs into the RT and qPCR reactions, respectively. Inhibition occurred in the European control plasma with 7 μl RNA. Graph shows the result of single reactions.


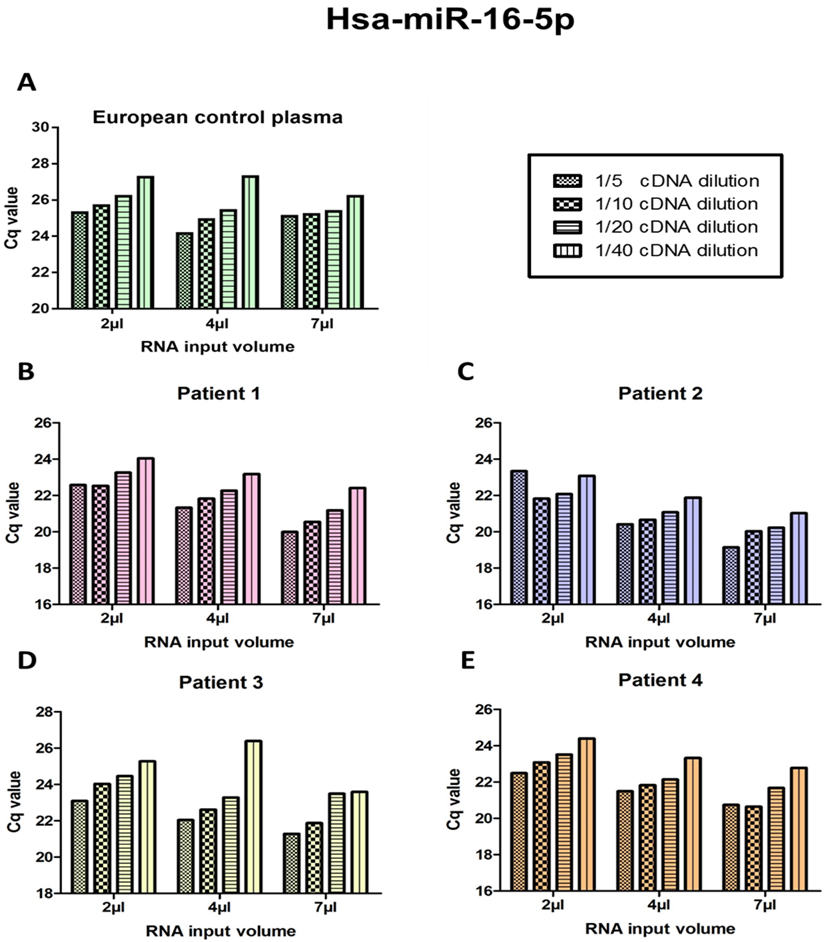


**Supplementary Fig. S4.** **Plasma reference and spike-in miRNA controls.**

**A.** The plasma endogenous control miRNA hsa-miR-16-5p was detected in all plasma samples with no significant differences between groups. Differences in hsa-miR-16-5p within the IVM and IVM+DOXY groups were also non-significant over time. Significant differences within the DOXY group (Friedman’s test: P = 0.0191) was detected between months four and 12 (Dunn’s post hoc test: P < 0.05). The mean of duplicate reactions for individual samples, and the ± SEM for each treatment group at each time point is shown.

**B.** The spike-in miRNA UniSp5 was present in all plasma samples, and similarly detected between and within groups over time. The average of two replicates for each individual, and the ± SEM for treatment groups at each time point are displayed. SEM, standard error of the mean; T0, baseline; T4, month four; T12, month 12; T21, month 21.

**
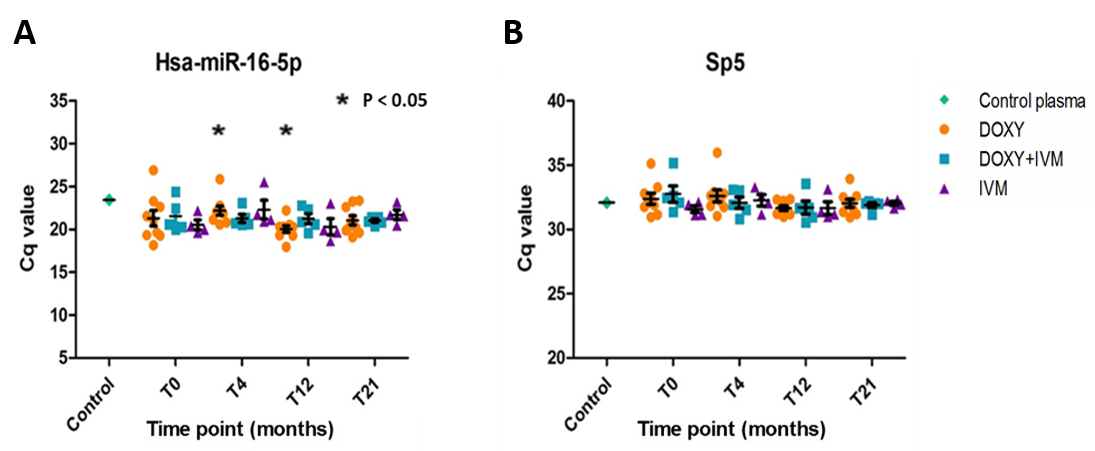
**

**Supplementary Fig. S5.** **Assay validation for *O. volvulus* O-150 DNA and endogenous plasma control GAPDH.**

**A.** Efficiency and precision of the O-150 assay was tested over five 1:2 dilutions of *O. volvulus* DNA. The average of three reactions and ± SD per dilution is shown. The overall inter- and intra-assay coefficient of variation (CV) was 0.69% and 0.04%, respectively. The average (± SD) efficiency of the qPCR across the three experiments was 97.4% (7.05).

**B.** Efficiency and precision of the GAPDH assay was tested over five 1:2 dilutions of plasma DNA. The average of three reactions and ± SD per dilution is shown. The overall inter- and intra- assay CV was 1.46% and 0.19%, respectively. The average (± SD) efficiency of the qPCR across the three experiments was 110.7% (11.54).

**
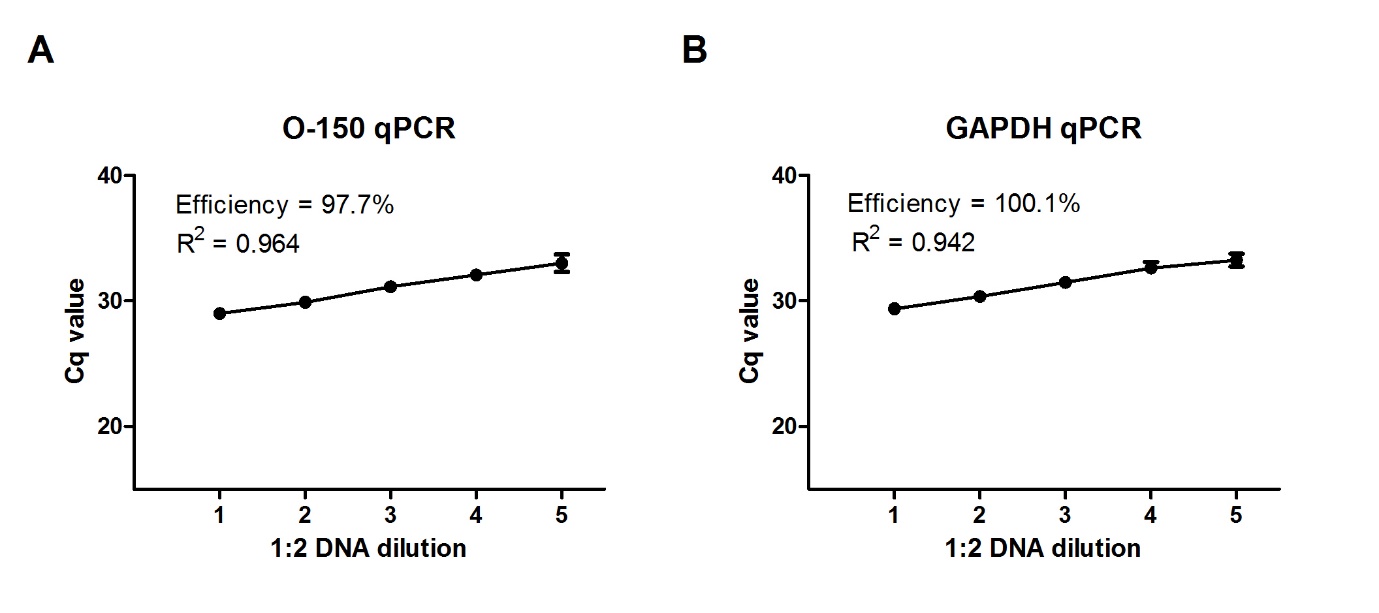
**

**Supplementary Fig. S6. Plasma reference and spike-in DNA controls.**

**A.** The plasma reference DNA marker GAPDH was detected in all plasma samples. There were no significant differences in GAPDH between or within groups over time. Graph shows the mean of two replicates for individual samples, and the ± SEM for each treatment group at successive time points.

**B.** The viral spike-in PhHV-1 was detected uniformly in all plasma samples. The mean of two replicates for individual samples, and the ± SEM for each treatment group at each time point are shown. T0, baseline; T4, month four; T12, month 12; T21, month 21.


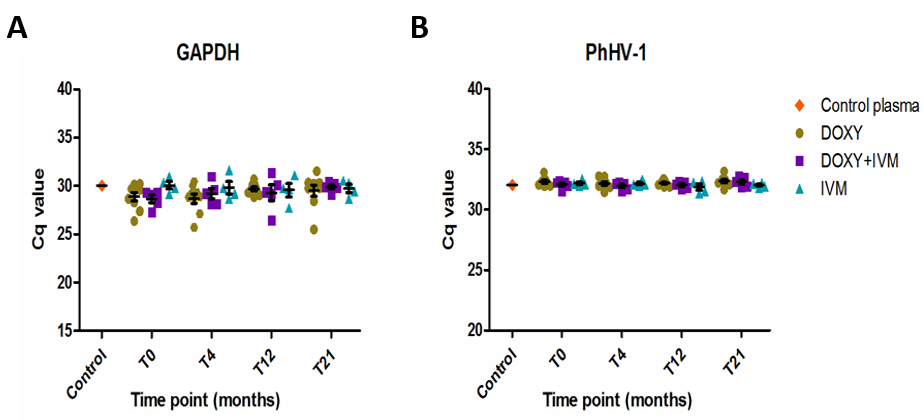


**Supplementary Table S3. Bioinformatic search for novel *O. volvulus* DNA repeat sequences.**

Summarised output of bioinformatic analysis for repeats in the *O. volvulus* genome. Newly identified repeat families specific for *O. volvulus* are considered as ‘unclassified’ repeats in this table.

SINEs, short interspersed nuclear elements; LINEs, long interspersed nuclear elements; LTR, long terminal repeats.

| **Repeat class** | **Total number** | **Nucleotide coverage** | **O. volvulus % genome coverage** |
| --- | --- | --- | --- |
| **Retroelements** | 5,368 | 1,023,174 | 1.07 % |
| SINEs | 80 | 5,372 | 0.01 % |
| Penelope | 259 | 21,668 | 0.02 % |
| LINEs | 2,098 | 179,252 | 0.19 % |
| LTR elements | 3,190 | 847,550 | 0.88 % |
| **DNA transposons** | 5,372 | 443,199 | 0.46 % |
| **Unclassified** | 34,730 | 6,923,829 | 7.19 % |
| **Simple repeats** | 61,799 | 2,486,882 | 2.58 % |
| **Low complexity** | 15,337 | 770,131 | 0.80 % |

**Supplementary Table S4. DNA repeat family targets identified in the *O. volvulus* genome.**

Bioinformatic searches for repeat sequences in the *O. volvulus* genome identified several repeat families predicted to occur more than O-150. The number of predicted occurrences, the contig locations and the consensus sequences are detailed for five highly abundant families (families A, B, C, D, E) and an additional five families (families F, G, H, I, J) located on contigs shared with the O-150 repeat.

**Supplementary Table S5. Detection of O-150 and novel DNA repeat families in clinical plasma samples.**

Cq values for O-150 and two candidate novel repeat families, Family A-III and Family G-II, evaluated by qPCR with trial patient plasma DNA. Empty cells denote no amplification.

| **Patient** | **qPCR replicate** | **O-150** | **Family A-III** | **Family G-II** |
| --- | --- | --- | --- | --- |
| a.2 | 1 | 38.65 |  |  |
|  | 2 |  |  |  |
| a.6 | 1 | 37.45 |  | 37.46 |
|  | 2 |  |  |  |
| a.7 | 1 | 33.70 | 34.67 | 36.31 |
|  | 2 | 32.24 |  | 35.76 |
| a.9 | 1 | 37.13 |  |  |
|  | 2 |  |  |  |
| b.1 | 1 |  |  |  |
|  | 2 |  |  |  |
| b.2 | 1 |  |  |  |
|  | 2 |  |  |  |
| b.4 | 1 | 35.50 |  |  |
|  | 2 |  |  |  |
| c.3 | 1 |  | 38.05 | 35.05 |
|  | 2 |  |  |  |
| c.4 | 1 |  |  | 35.76 |
|  | 2 |  |  |  |
